# Specified Certainty Classification, with Application to Read Classification for Reference-Guided Metagenomic Assembly

**Published:** 2021-09-13

**Authors:** Alan F. Karr, Jason Hauzel, Prahlad Menon, Adam A. Porter, Marcel Schaefer

**Affiliations:** Center Mid-Atlantic, Fraunhofer USA; Center Mid-Atlantic, Fraunhofer USA; Center Mid-Atlantic, Fraunhofer USA; Center Mid-Atlantic, Fraunhofer USA; Center Mid-Atlantic, Fraunhofer USA

**Keywords:** metagenomics, read classification, classifier, uncertainty quantification, Bayesian analysis, posterior probabilities

## Abstract

Specified Certainty Classification (SCC) is a new paradigm for employing classifiers whose outputs carry uncertainties, typically in the form of Bayesian posterior probabilities. By allowing the classifier output to be less precise than one of a set of atomic decisions, SCC allows all decisions to achieve a specified level of certainty, as well as provides insights into classifier behavior by examining all decisions that are possible. Our primary illustration is read classification for reference-guided genome assembly, but we demonstrate the breadth of SCC by also analyzing COVID-19 vaccination data.

## I. Introduction

Classifiers are ubiquitous in bioinformatics, with applications ranging from genomics—the primary setting here—to radiology. A classifier, developed from labeled training data, places new objects into one of a finite number of classes. Nearly all extant classifiers, including those based on deep learning models in artificial intelligence (AI), provide little or no information regarding associated uncertainties [8]. Even more rarely is there control over those uncertainties. *Specified Certainty Classification (SSC)*, introduced here, gives analysts control over the certainty levels of the output of classifiers based on Bayesian posterior probabilities.

The distinguishing characteristics of SCC are that: (1) All data point-specific decisions attain a specified level of certainty, denoted by *β* below; (2) SCC allows the analyst to investigate *all decisions that can be made* rather than *what decisions are made* for specific settings of the threshold; and (3) SCC allows investigation of the thresholds at which data points lose precision. We discuss each briefly here and in detail below.

To achieve specified certainty, SCC permits some decisions to be less precise than others. Specifically, if A is the set of atomic decisions, then rather than restrict output to be *elements* of A, SCC allows a decision to be any nonempty *subset* of A. To illustrate, in §IV, where the problem is to classify (short) DNA sequence reads as arising from one of three “reference” genomes—in this case, an adenovirus genome and two coronavirus genomes, A={Adeno, COVID, SARS}, but some reads may be classified as “COVID or SARS,” or even “Adeno or COVID or SARS.” Loss of precision occurs when the decision is compound rather than atomic.^1^

By investigating all decisions, SCC embodies the paradigm in [12], where inpatient and outpatient databases from a major healthcare system in the U.S. were used to compare the performance of a number of software packages for record linkage. That paper demonstrated that some packages have dramatically richer decision-making capability than others. In turn, this capability facilitates construction of receiver operating characteristic (ROC) curves [7] that quantify the tradeoffs between false negatives (records that should have been linked, but were not, which is conventionally interpreted as bias) and false positives (records that were linked but should not have been, which is interpreted as noise), and enables construction of ensemble methods that outperform all the individual methods. Here, we are able to construct ROC-like curves for SCC that quantify the increase in accuracy resulting from loss in precision. See §IV.

Transition points are particularly insightful for small datasets: in §V we apply SCC in a healthcare context, to investigate COVID-19 vaccination rates in U.S. states.

The major contributions of the paper are formulation of SCC and demonstration of its power in two very different bioinformatics/biomedicine contexts. The paper is organized as follows. We present SCC concretely, via the read classification problem. §II describes the experiment we conducted, and §III introduces the Bayesian classifier, which is based on triplet distributions [10] for the reference genomes. §IV presents SCC and articulates its properties. To demonstrate broad applicability, §V applies SCC to COVID-19 vaccination data. Conclusions appear in §VI.

## II. Experimental Context

Our three reference genomes are an adenovirus (sometimes Adeno) genome of length 34,125, downloaded with the read simulator Art, a SARS-CoV-2 genome of length 29,926 contained in a coronavirus dataset downloaded from National Center for Biotechnology Information (NCBI) in November of 2020,^2^ which we call COVID, and a severe acute respiratory syndrome (SARS) genome of length 29,751 from the same NCBI database.

The Mason_simulator read simulator [9] was used to simulate Illumina^3^ reads of length 101 from each of the three genomes, with approximately 6X coverage. The numbers of reads in our dataset are 1966, 1996 and 1907, respectively, which total to 5869. Parameters of Mason_simulator were set at their default values. The Mason_simulator introduces errors in the form of transpositions (SNPs), indels and undetermined bases, which following convention appear in the simulated reads as “N” and must be accommodated in computation of likelihood functions.

The read classification problem is to determine the source genome for each of the 5869 reads. All analyses reported below were performed using R [15].

## III. The Bayesian Classifier

As in any Bayesian analyses, there are three components. The first is the reads themselves—the data. The second is the prior probabilities—for each read *R*, a probability distribution *π*_*R*_ over A={Adeno, COVID, SARS}, the set of atomic decisions. The third component comprises a likelihood function for each genome, calculated from the triplet distributions of nucleotides (bases) using the methodology outlined below and investigated at length in [10]. We refer to those as models, for which the three genomes comprise the training data. The likelihood functions are denoted by *L*_*A*_(·), *L*_*C*_(·) and *L*_*S*_(·) for adenovirus, COVID and SARS.

To illustrate for adenovirus, the triplet distribution is the probability distribution *P*_3_(·|*A*) on the 64-element set of all ordered triplets selected from the nucleotide alphabet {*A*, *C*, *T*, *G*} given by

(1) $$P_{3}\left(b_{1}b_{2}b_{3}|A\right)=\text{Prob}\left\{A(K:[K+2])=b_{1}b_{2}b_{3}\right\},$$

where *A*(*k* : [*k* + 2]) is the length 3 substring of the adenovirus genome *A* commencing at *k* and *K* is chosen at random from 1,…,34,123 (where the last triplet begins). Computation of *L*_*A*_(·) from *P*_3_(·|*A*) is accomplished by means of the analogous pair distribution *P*_2_(·|*A*) and the second-order Markov transition matrix

(2) $$T_{3}\left(b_{1},b_{2},b_{3}|A\right)=\text{Prob}\left(A(k+2)=b_{3}|A(k)=b_{1},A(k+1)=b_{2}\right).$$

The care necessary to handle Ns—present but undetermined bases—is exercised most easily with the pair distribution–transition matrix formulation.

In [10], all three components of the Bayesian paradigm are varied, and their contributions to decisions resolved The actual, error-containing reads can be replaced by error-free reads (available from the Mason_simulator), or degraded using the Mason_variator, as in [11]. The priors can be informative, as below, or uniform over A, or even incorrect in the sense of omitting one genome [10]. Quality of the models can be reduced by degrading the reference genomes using the Mason_variator.

The Bayesian analysis itself is straightforward: we use Bayes’ theorem and the three likelihoods to calculate posterior probabilities over A. Specifically, for x∈A, the posterior probability of *x* for read *R* is

(3) $$p(x|R)=\frac{\pi_{R}(x)L_{x}(R)}{\pi_{R}(A)L_{A}(R)+\pi_{R}(C)L_{C}(R)+\pi_{R}(S)L_{S}(R)}.$$

**Fig. 1.**
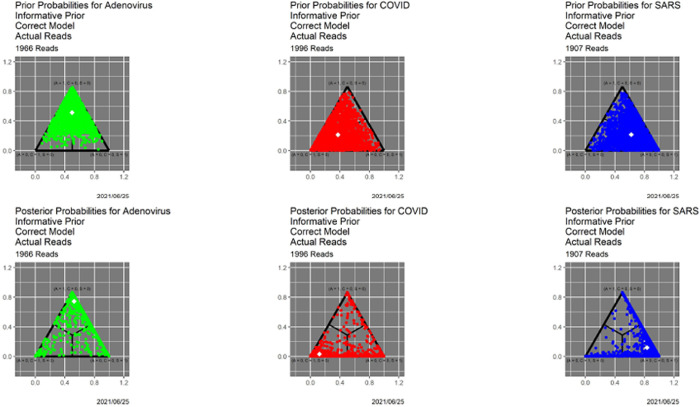
Prior and posterior probabilities for the base case of actual reads, informative prior distributions and correct triplet distribution-based models. *Top left:* prior probabilities for reads from adenovirus. *Top middle:* prior probabilities for reads from COVID. *Top right:* Prior probabilities for reads from SARS. *Bottom left:* posterior probabilities for reads from adenovirus. *Bottom middle:* posterior probabilities for reads from COVID. *Bottom right:* Posterior probabilities for reads from SARS. White symbols in each plot are centroids.

Figure 1 shows the prior and posterior probabilities for our base case of informative priors,^4^ the actual reads, and the correct models based on the triplet distribution likelihood functions from the three genomes. Because similar figures follow below, it is worthwhile to discuss it in some detail. First, there is one point for each read. Three-dimensional probabilities (barycentric coordinates) are represented in Cartesian coordinates as points in a two-dimensional simplex—an equilateral triangle. Pure adenovirus, in the sense that Prob(Adeno|*R*) = 1, is the top vertex, pure COVID is the lower left vertex, and pure SARS is the lower right vertex. Because this is an experiment and we know the sources of the reads, there is a separate display for each source—adenovirus at the left, COVID in the center and SARS at the right. The upper three panels show prior probabilities, while the lower three panels show posterior probabilities. The green/red/blue coloring encoding read source is redundant but useful. The white dot in each graphic is the centroid of the probabilities it contains. The interior black lines, which are sometimes not visible, are the decision boundaries for the classifier that maximizes posterior probability over A—the *MAP classifier*.

Table I shows the confusion matrix for the MAP classifier. The performance is decent: the correct classification rate—calculable because this is an experiment with known ground truth—is 86.35%.

Many analyses would simply stop here, without reporting uncertainties, let alone making use of them. The bottom panel in Figure 1 demonstrates unequivocally that *all decisions are not equal*, even when they are correct. In particular, for all three genomes, there are correctly classified reads that lie near the center of the triangle, meaning that they are close calls. To illustrate with an extreme case, if *p*(·|*R*) = (0.34, 0.33, 0.33), *R* will be classified as adenovirus, even though many analysts might feel uncomfortable with this. Going one step further, the MAP classifier does not distinguish *R* from *R*′ for which *p*(·|*R*′) = (0.99, 0.005, 0.005).

## IV. Specified Certainty Classification

**Fig. 2.**
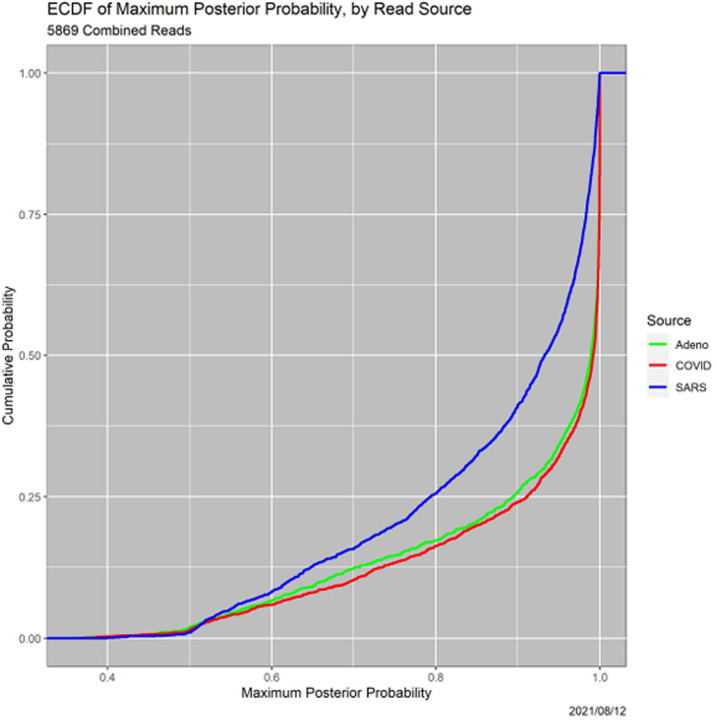
ECDFs of certainty of MAP classifications, by read source.

One can go beyond common practice by at least reporting the uncertainties of the MAP classifications, for instance, summarizing them with the empirical cumulative distribution functions (ECDFs) in Figure 2. This figure establishes that many MAP decisions are not very certain. More than 15% of all reads, and more than 25% of the SARS reads, are classified with certainty less than 80%. (Since the *p*(Adeno|*R*) + *p*(COVID|*R*) + *p*(SARS|*R*) = 1 for each *R*, the maximum posterior probability cannot be less than 1/3.) In some circumstances, this may not be acceptable. Moreover, decisions for SARS are clearly, and statistically significantly, less certain than those for adenovirus or COVID. This distinction recurs throughout the paper.

**Fig. 3.**
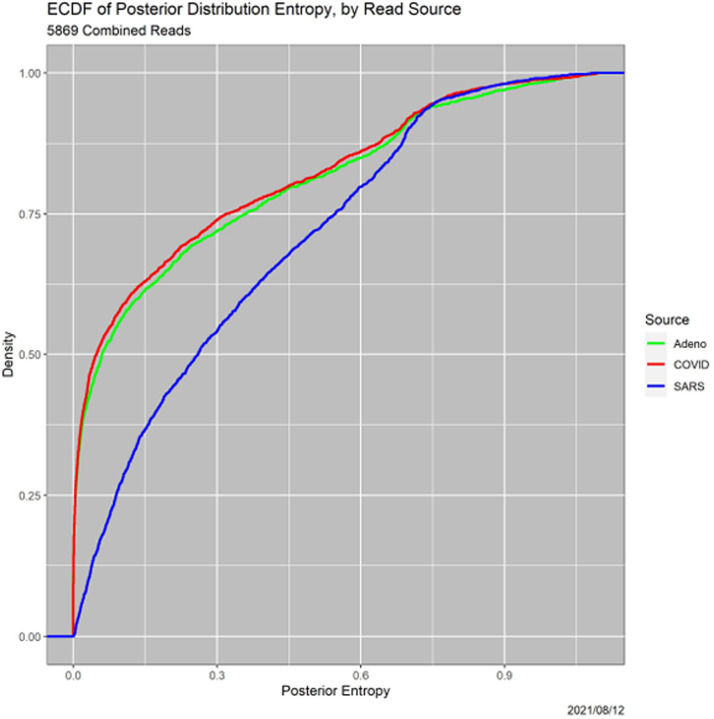
ECDFs functions of posterior entropies, by read source.

Complementarily, Figure 3 shows ECDFs of the entropies of the posterior distributions, again broken down by read source. The lower the entropy, the more definitive the MAP classification.^5^ Three features of this figure are striking. First are the secondary modes, for all three sources, at approximately 0.7, which we do not pursue. The second is that the SARS ECDF differs dramatically from those for adenovirus and COVID, which do not differ significantly from one another. The two-sided *p*-values for Kolmogorov–Smirnov tests are 0 for COVID–SARS and 0.120 for COVID–adenovirus, Third and most important for this paper, for all three genomes there is considerable mass on values of entropy that do not represent clear-cut decisions by the MAP classifier.

### A. Formulation

In response to these concerns, SCC inverts and broadens the perspective. Instead of using MAP and having to live with the resultant uncertainties, the analyst specifies the desired (or required) level of certainty, and SCC allows the classifier to produce less precise results that achieve it. Rather than forcing the classifier output to be one of ‘Adeno’, ‘COVID’ or ‘SARS,’ the atomic decisions, SCC allows the classifier output to be *any* nonempty subset of A, as long as the certainty level exceeds the specified threshold. Mathematically, the allowable classifier outputs under SCC are the power set of A, excluding the empty set ∅:

(4) P(A)={{Adeno},{COVID},{SARS},{Adeno, COVID},{Adeno, SARS},{COVID, SARS},{Adeno, COVID, SARS}}.

Here lies one computational challenge: the number of elements in P(A) is $2^{\left|A\right|}$, where |*S*| is the cardinality of the set *S*. However, in applications, only the “occupied” elements of this set need to be enumerated or stored, which can be done using trees.

Computationally, SCC is straightforward. Given the certainty level *β*, for each read *R*, sort the posterior probabilities *p*(Adeno|*R*), *p*(COVID|*R*) and *p*(SARS|*R*) in decreasing order, leading to a data structure

(5) $$D=\left[\begin{array}{ll} A_{1} & p\left(A_{1}|R\right)\\ A_{2} & p\left(A_{2}|R\right)\\ A_{3} & p\left(A_{3}|R\right) \end{array}\right],$$

where (*A*_1_, *A*_2_, *A*_3_) is a permutation of A and

$$p\left(A_{1}|R\right)\geq p\left(A_{2}|R\right)\geq p\left(A_{3}|R\right).$$

Let

$$K=\text{min }\left\{j:p\left(A_{1}|R\right)+\ldots p\left(A_{j}|R\right)\geq \beta \right\};$$

then the decision of *R* is {*A*_1_, …, *A*_*K*_}. Note that β=1/|A| produces the MAP classifier, because *p*(*A*_1_|*R*) ≥ *β* for all *R*. With *β* specified, therefore,

- Some reads will be classified as ‘Adeno’, as ‘COVID’ or as ‘SARS,’ meaning that with posterior certainty at least *β* the read arises from exactly one of the three genomes.
- Other reads will be classified as ‘Adeno-or-COVID,’ ‘Adeno-or-SARS’, or ‘COVID-or-SARS,’ meaning that with certainty *β* they can only be said to have arisen from one of two genomes. This is the “loss of precision” referred to in §I.
- Still other reads will be classified as ‘Adeno-or-COVIDor-SARS,’ the equivalent of “unclassifiable.”

Below, we refer to these as decisions of Precision 1, 2 and 3, respectively.

**Fig. 4.**
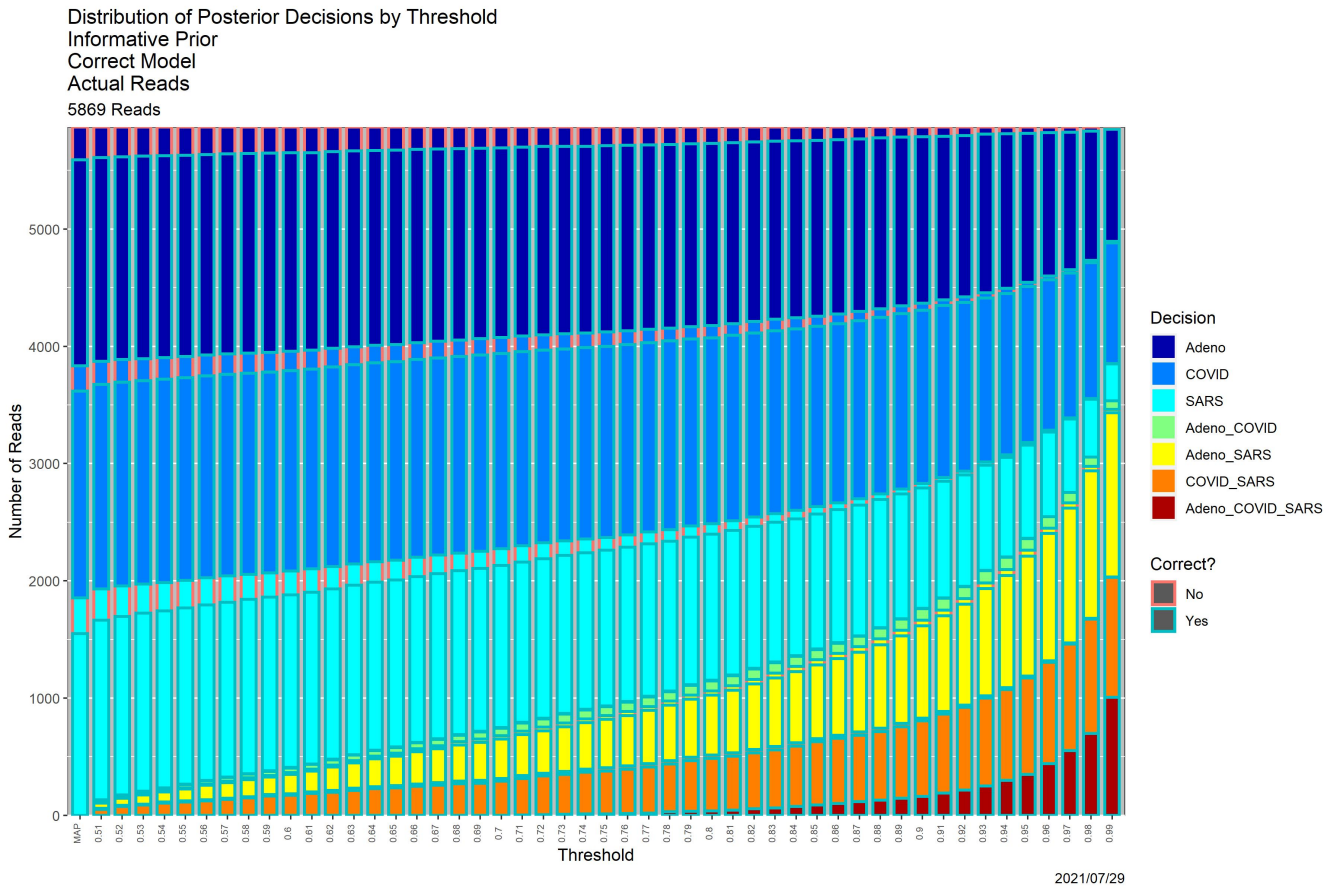
SCC output for the posterior probabilities in Figure 1.

Figure 4, which is in some sense our “takeaway message,” shows the result of applying SCC to the reads dataset, using the posterior probabilities appearing in Figure 1. The horizontal axis contains thresholds of 0.33 (as noted above, the MAP estimator) and 0.51, …, 0.99. The bar for each threshold shows the distribution of classifier output for that threshold, split in two ways. Color of the bar encodes the decision, and color of the surrounding box indicates whether the decision is correct. “Correct” no longer means an exact match. For instance, if the source of read *R* is adenovirus, the decision is correct if the classifier output for *R* is any one of ‘Adeno’, ‘Adeno-or-COVID,’ ‘Adeno-or-SARS,’ or ‘Adeno-or-COVID-or-SARS.’ While ground truth is required to determine correctness (box color), it is not required to determine the distribution of decisions (bar color).

### B. Tradeoffs

Qualitatively, Figure 4 shows exactly the behavior we expected. As the threshold increases, precision decreases. For low thresholds (at the left), Precision 1 (a single genome) decisions predominate, and while some of these remain for high thresholds (at the right) most decisions become Precision 2 or 3. The same can be seen more quantitatively by comparing the ”Posterior Probabilities” columns in Tables III (*β* = 0.80) and II (*β* = 0.99) below. There is no universally correct choice of threshold. Indeed, SCC, as in [12], is to empower the user by illuminating the consequences of using it.

**Fig. 5.**
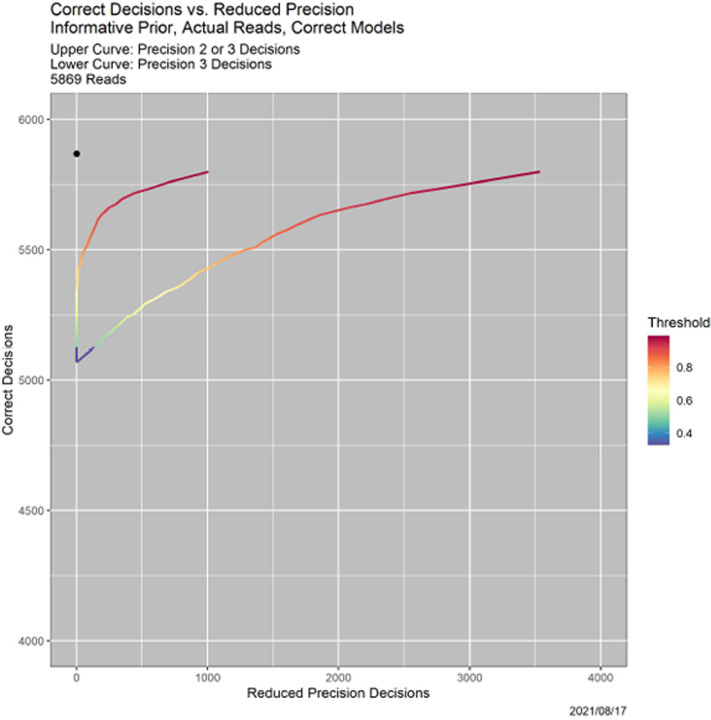
Tradeoff of correctness and precision for the example in Figure 4. *Lower curve:* incorrect decisions vs. Precision 2 decisions. *Upper curve:* Incorrect decisions vs. Precision 2 or 3 decisions.

There are also more subtle behaviors. Figure 5 shows how SCC trades off precision and correctness. There are two curves, each parameterized by the threshold *β*. The *y*-axis is always the number of correct reads. The *x*-axis is the number of “less precise reads,” which is different for the two curves, and explained momentarily. As for ROC curves, optimality is the black dot at the upper left, representing 5869 correct reads and no less precise reads. For the upper curve, less precise means Precision 2 or 3. For the lower curve, it means Precision 3 alone.

### C. Classifier Behavior

**Fig. 6.**
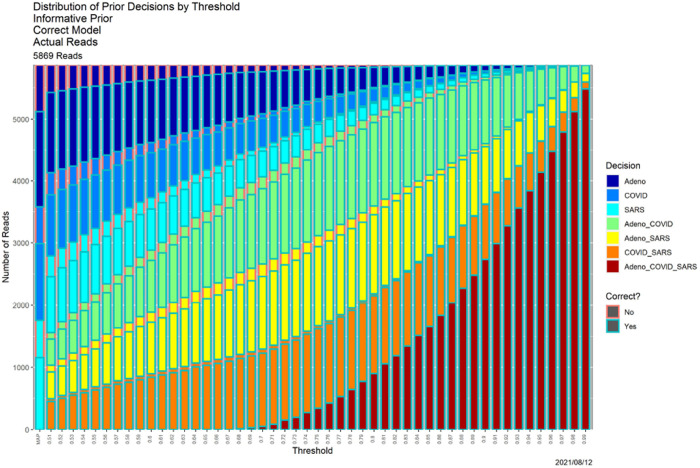
SCC output for the prior probabilities in Figure 1.

SCC also illuminates the classifier itself. First, SCC can be applied to prior probabilities. Figure 6 contains SCC results for the prior probabilities for the base case. The Bayesian computation in (3) transforms the decision structure in Figure 6 into that in Figure 4. Uniformly for all thresholds, using posterior probabilities leads to larger numbers of high precision decisions than using the priors alone. At an extreme, for *β* = 0.99 (the far right in both figures) using prior probabilities, nearly all reads are classifiable only as ‘Adeno-or-COVID-or-SARS,’ whereas when posterior probabilities are employed, only approximately 15% are. Table II provides a detailed comparison. This table quantifies how dramatically decisions are improved by adding data and models to the prior probabilities. Even for the much less conservative threshold of *β* = 0.80, similar behavior obtains, as shown in Table III.

**Fig. 7.**
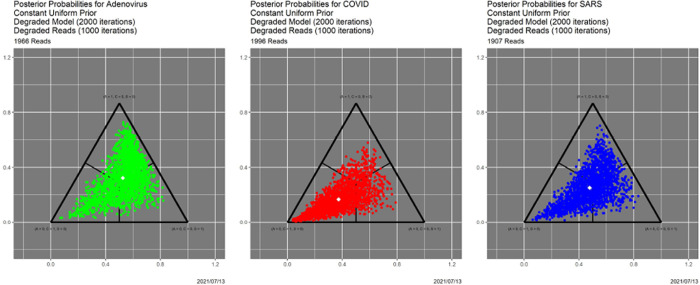
Posterior probabilities for the “worst of all possible worlds” scenario.

In [10] we explore in depth the effects of varying the prior, the quality of the read data, and the quality of the models, which reflects the quality of the training data. Without replicating the granularity of that exploration, we show here how SCC delivers complementary insights. The “worst of all possible worlds” scenario in [10] is one in which all priors are uniform over A, reads are degraded by 1000 iterations of the Mason_variator and the training data underlying the likelihood functions are degraded by 2000 iterations of the Mason_variator on the actual genomes, before calculating the triplet distributions and likelihood functions.. The posterior probabilities appear in Figure 7. Only the most cursory visual comparison with Figure 1 is needed to know that something is seriously wrong. Indeed, this figure more closely resembles Figure 6 than Figure 1. In other words, the starting point for the base case does not differ dramatically from the ending point for the “worst of all possible worlds” scenario.

**Fig. 8.**
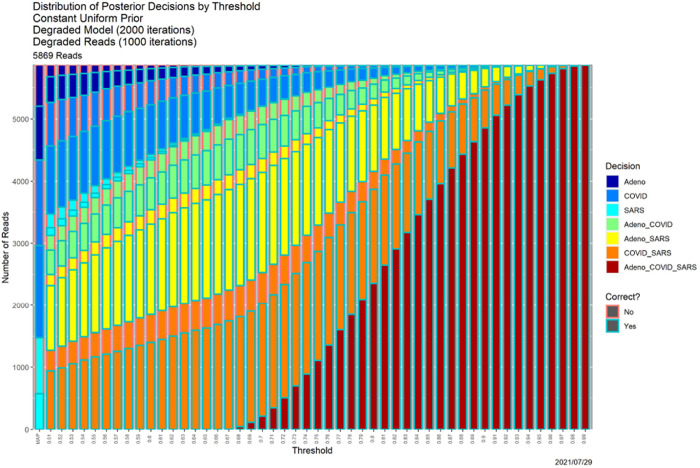
SCC output for the “worst of all possible worlds” posterior probabilities in Figure 7.

Figure 8 shows that the SCC consequences are similarly deleterious. Again choosing *β* = 0.80 as exemplar, Table IV contains the associated decisions; it should be compared to the “Posterior Probabilities” column of Table III.

### D. Transition Points

**Fig. 9.**
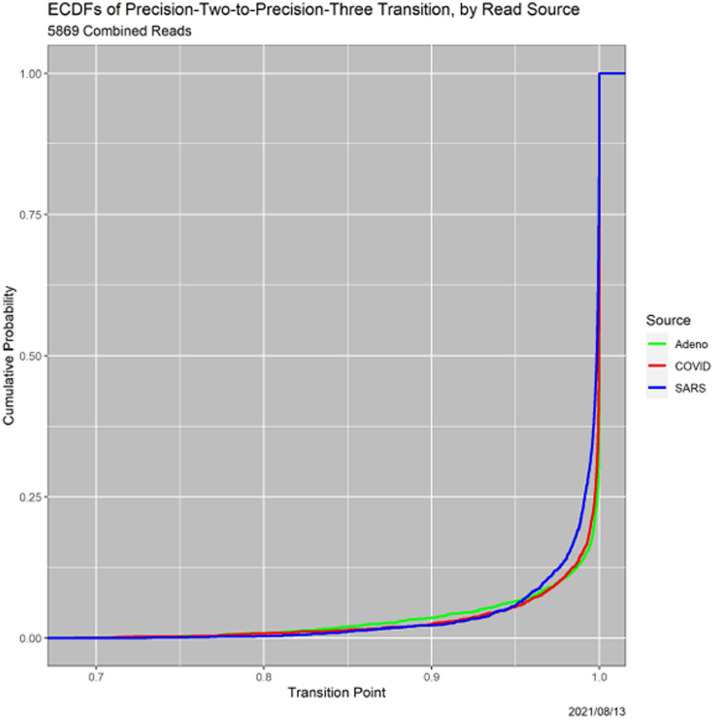
ECDFs of Precision-2-to-Precision-3 transitions, by read source.

A third pathway to insight, which arose in the example in §V (see Table VIII), is to examine the thresholds at which loss of precision occurs. Each read undergoes two loss-of-precision transitions, for instance, from ‘Adeno’ to ‘Adeno-or-COVID’ and then to ‘Adeno-or-COVID-or-SARS.’ ECDFs of the transitions are informative. Since the Precision-1-to-Precision-2 transition is identical to the certainty of the MAP estimator, Figure 2 contains the ECDFs of the Precision1-to-Precision-2 transitions. Figure 9, is analogous, but for Precision-2-to-Precision-3 transitions. Notably, the SARS versus Adeno and COVID gap for Precision-1-to-2 transitions is diminished significantly, although it does not vanish and remains statistically significant.

### E. Collective Certainty

This material is narrow but important. The certainty levels promised by SCC apply to each data point (read, in this case) individually, not to the collective of decisions. There is an extensive literature on multiple testing in statistics [5, 18]. Whether SCC decisions can be treated in the same way as the results of hypothesis tests is not clear at this point. Bonferroni corrections, meaning essentially that the decisions are treated as independent, may lead extremely small estimates of collective certainty, especially for large datasets. Alternatives such as false discovery rates [3] may be more promising. An initial example appears in §V, where the effect of increasing the threshold is unmistakeable.

## V. Application to COVID-19 Vaccination Data

The SCC paradigm works in any context where the classifier output consists of (posterior) probabilities on a finite set A of atomic decisions. To illustrate, in this section we analyze state-level COVID-19 vaccination rates as they relate to the outcome of the 2020 election (Democratic or Republican, again by state). The analysis data consist of 51 state proportions *V* (*S*) of the entire population who were fully vaccinated as of July 31, 2021, which were downloaded from the Centers for Disease Control and Prevention (CDC) website.^6^ To give a sense of the data, the fully-vaccinated proportions vary from 34.3% (AL) to 67.5% (VT).

### A. Mixture Model

**Fig. 10.**
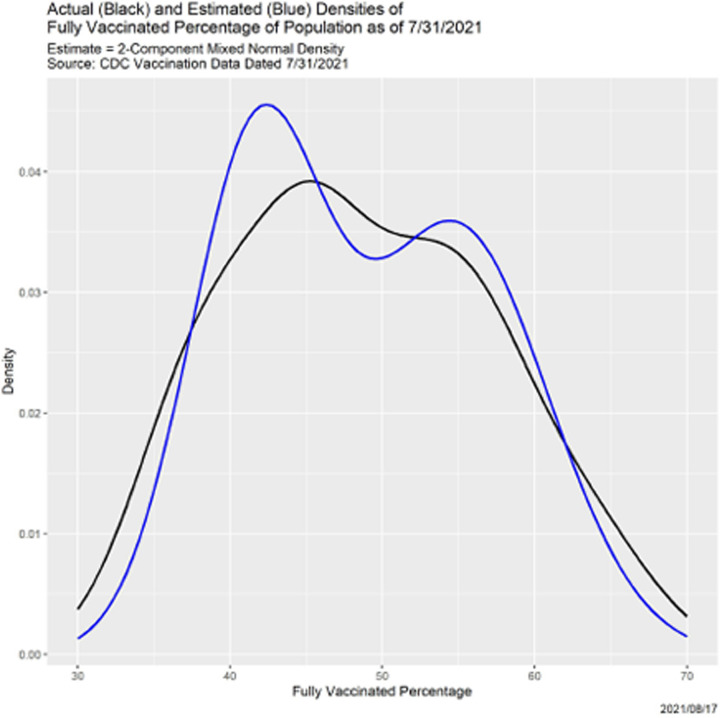
Actual and estimated densities of fully COVID-19 vaccinated percentages as of 7/31/2021.

Figure 10 shows in black the density function of the 51 fully vaccinated percentages. (The blue curve will be explained momentarily.) There is clear evidence of bimodality, so it is natural to model this density as a two-component mixture of normal distributions. We did so using the mixtools package in R [2], whose algorithm derives from [13]. Table V shows the weights, means and standard deviations of the two components of the resulting mixture model. The blue line in Figure 10 is the fitted density. The fit is better than Figure 10 may seem to suggest: of 1000 bootstrapped samples of size 51 from the mixed normal density, only three led to rejection of the hypothesis that the two densities are identical.

**Fig. 11.**
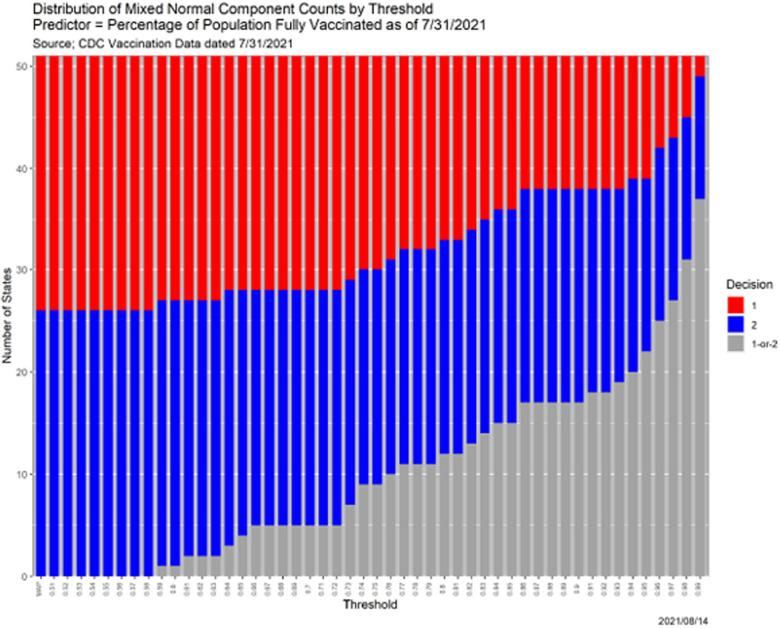
SCC results for 2-component mixed normal model of state-level percentages of fully COVID-vaccinated population.

Often mixing arises from an unobserved, or at least unmodeled, *latent variable* [4] taking as many possible values as there are mixture components—in this case, two. SPOILER ALERT: as the colors in Figure 11 suggest, we hypothesize that the latent variable measures the outcome of the 2020 Presidential election, which we denote by *O*(*S*) ∈ {*D*, *R*}. Note that this interpretation of the mixture model is consistent with modern thinking regarding causality [14], in the sense that the potential causality is in the right direction.^7^ The relationship might still, of course, not be causal: voting and vaccination could both result from common underlying causal factors such as age, education, income or race.

### B. Application of SCC

Returning to the mixture model, reflecting the unobserved nature of latent variables, many fitting methods, including mixtools, employ the iterative EM (expectationmaximization) algorithm [6]. The primary outputs, in consequence, are posterior distributions over the components for each data point. Mixture models are, therefore, natural candidates for SCC, and Figure 11 contains the results of applying it to the case at hand.

The qualitative interpretations are similar to those in §IV. With two outcomes, the MAP estimator corresponds to *β* = 1/2. Figure 11—more precisely, the numerical values underlying it—is more informative scientifically that any single set of predictions. For instance, contrast with §IV, there are states that are predicted to be a single component even when *β* = 0.99. Two states, AL and MS, are always in Component 1, while CT, MA, MD, ME, NH, NJ, NM, NY, OR, RI, VT, and WA (12 states) are always in Component 2. Only once *β* ≥ .59 is any state not placed in a single component; from Table VIII, that state is SD.

Figure 11 does not, *per se*, dictate what threshold should be chosen in a given context. It does allow an analyst to understand his or her choice of a threshold in light of alternatives. To illustrate, there is no reason to choose *β* ∈ [.66, .71] because the results are identical to those for *β* = .72. Figure 11 does not even dictate that a single threshold must be chosen, because considering all thresholds is more informative.

As intimated, the colors in Figure 11 were selected deliberately. Table VI is a cross-tabulation of state-level results in the 2020 Presidential election with the MAP component assignments. Only 6 states are “mis-classified:” AZ, GA, and NV voted Democratic but are in Component 1, while FL, IA and NE voted Republican but are in Component 2.^8^ Illustrating the kinds of insights SCC facilitates, Table VII shows the same information for *β* = 0.80 and 0.98, the minimum threshold for which there are no errors.

Because there are only 51 data points and only one loss-ofprecision transition for each, loss-of-precision for the analysis in Figure 11 can be enumerated, resulting in Table VIII. Each state undergoes one transition as *β* increases, from either ‘1’ or ‘2’ to ‘1-or-2;’ these are the values in the table.

**Fig. 12.**
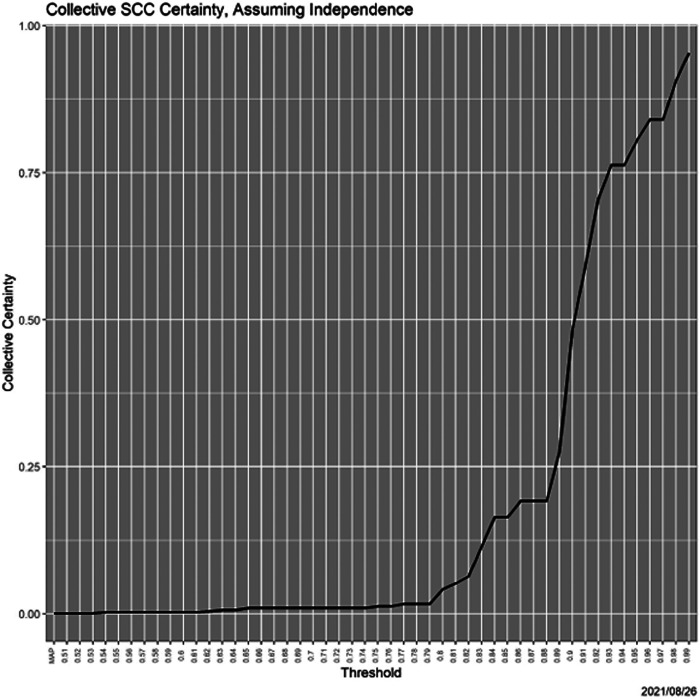
Collective certainty as a function of threshold.

Finally, returning to collective certainty (§IV-E), Figure 12 shows the dramatic increase in collective certainty relative under the heuristic and *ad hoc* scenario of independence of decisions over states.

## VI. CONCLUSION

SCC empowers analysts and decision makers. First and foremost, it creates control of the level of certainty in classifier output by allowing some decisions to be less precise than others. Contrastingly, MAP classifiers classify every data point with Precision 1; SCC shows the potentially negative implications of doing so. SCC also provides unprecedented insight into the behavior of classifiers and the consequences with respect to making decisions. Moreover, SCC allows those of us who develop tools to be scientifically agnostic about their use: SCC does not make decisions, but instead provides an explainable basis for making principled decisions. Finally, SCC is a step in the direction of interpretability, a major issue for AI [1].

Much remains to be done. Many classifiers, especially AIbased deep learning models and trees, do not currently provide the posterior probabilities required by SCC. One potential path is that warned against in [1], namely developing approximations that do produce uncertainties.^9^

Extension is also needed to cases where our “allow all subsets of the space of atomic decisions” is not feasible.^10^ The necessary infrastructure is that the set of allowable compound decisions must be partially ordered by set inclusion.

## Figures and Tables

**TABLE I:** Confusion matrix for the MAP classifier for the base case. Rows are read sources; columns are MAP decisions. The correct classification rate is 86.35%.

	Decision			
Source	Adeno	COVID	SARS	Sum
Adeno	1757	74	135	1966
COVID	64	1762	170	1996
SARS	214	144	1549	1907
Sum	2035	1980	1854	5869

**TABLE II:** SCC results for threshold *β* = 0.99 using prior and posterior probabilities that appear in Figure 1.

	Probabilities	
Decision	Prior	Posterior
Adeno	0	975
COVID	1	1042
SARS	1	316
Adeno-or-COVID	131	73
Adeno-or-SARS	139	1006
COVID-or-SARS	111	1025
Adeno-or-COVID-or-SARS	5486	1075

**TABLE III:** SCC results for threshold *β* = 0.80 using prior and posterior probabilities that appear in Figure 1.

	Probabilities	
Decision	Prior	Posterior
Adeno	363	1694
COVID	227	1689
SARS	208	1334
Adeno-or-COVID	1479	80
Adeno-or-SARS	1409	548
COVID-or-SARS	1280	475
Adeno-or-COVID-or-SARS	903	39

**TABLE IV:** SCC results for threshold *β* = 0.80 and the “worst of all possible worlds” scenario.

Decision	Number of Reads
Adeno	9
COVID	205
SARS	0
Adeno-or-COVID	330
Adeno-or-SARS	1166
COVID-or-SARS	1803
Adeno-or-COVID-or-SARS	2356

**TABLE V:** Parameters of the Mixed Normal Model.

Parameter	Component 1	Component 2
Weight *λ*	0.4678343	0.5321657
Mean *μ*	41.7228	54.8779
Standard deviation *σ*	4.447488	6.008174

**TABLE VI:** Cross-tabulation of MAP component assignments and state-level 2020 Presidential election outcomes.

2020 Election Outcome			
Component	Democratic	Republican	Sum
1	3	22	25
2	23	3	26
1-or-2	0	0	0
Sum	26	25	51

**TABLE VII:** *Top:* Cross-tabulation of component assignments for *β* = 0.80 and state-level 2020 Presidential election outcomes. *Bottom:* Cross-tabulation of component assignments for *β* = 0.98 and state-level 2020 Presidential election outcomes.

2020 Election Outcome			
Component	Democratic	Republican	Sum
1	2	16	18
2	21	0	21
1-or-2	2	9	12
Sum	26	25	51

2020 Election Outcome			
Component	Democratic	Republican	Sum
1	0	6	6
2	14	0	14
1-or-2	12	19	31
Sum	26	25	51

**TABLE VIII:** Transition points associated with Figure 11.

State	2020 Outcome	Loss of Precision	State	2020 Outcome	Loss of Precision
AK	R	73.68	MT	R	82.55
AL	R	99.05	NC	R	85.35
AR	R	98.50	ND	R	95.81
AZ	D	75.38	NE	R	72.24
CA	D	94.63	NH	D	99.87
CO	D	97.99	NJ	D	99.88
CT	D	100.00	NM	D	99.70
DC	D	98.45	NV	D	81.93
DE	D	94.31	NY	D	99.68
FL	R	65.37	OH	R	63.81
GA	D	97.25	OK	R	95.54
HI	D	96.47	OR	D	99.31
IA	R	73.32	PA	D	92.85
ID	R	98.07	RI	D	99.99
IL	D	60.48	SC	R	95.09
IN	R	83.15	SD	R	58.12
KS	R	76.20	TN	R	96.91
KY	R	72.79	TX	R	85.35
LA	R	98.31	UT	R	79.24
MA	D	100.00	VA	D	98.12
MD	D	99.91	VT	D	100.00
ME	D	100.00	WA	D	99.78
MI	D	64.16	WI	D	90.58
MN	D	97.07	WV	R	97.00
MO	R	93.62	WY	R	98.39
MS	R	99.01			

## Footnotes

1 Thus use of “precision” is not that in machine learning, where precision means the same as positive predictive value in biostatistics.

2 The link is https://www.ncbi.nlm.nih.gov/datasets/coronavirus/genomes/.

3 Illumina is a major manufacturer of instruments for genome sequencing; their technology is optical in nature; see https://www.illumina.com/.

4 These were simulated using Dirichlet distributions that are biased modestly, albeit not uniformly, toward the truth.

5 At the extreme, entropy is zero only for distributions concentrated at one point, and is maximized at the uniform distribution.

6 The vaccination data, updated weekly, are at https://covid.cdc.gov/coviddata-tracker/#vaccinations. Weekly state-level case and death counts are also available.

7 An alternative approach that estimated two densities for vaccination, one for states with *O*(*S*) = *D* and one for states with *O*(*S*) = *R*, would at the person level be highly suspect because it posits that vaccination causes voting behavior. Not only is that relationship retroactive in time, but also there is no scientifically plausible causal path.

8 Other analyses not reported here show these six states to be “vaccination outliers.” For instance, the high population of seniors in FL, most of whom are vaccinated, distorts an otherwise low vaccination rate.

9 An intriguing analogy is computer experiments [16, 17], in which Gaussian process priors are used to develop approximations to complex numerical models.

10 For example, if the atomic decisions are ages, the feasible compound decisions might be constrained to be age ranges, because a decision “age = 23-or-27-or-31” is nonsensical.
